# Robotic lower limb prosthesis design through simultaneous computer optimizations of human and prosthesis costs

**DOI:** 10.1038/srep19983

**Published:** 2016-02-09

**Authors:** Matthew L. Handford, Manoj Srinivasan

**Affiliations:** 1 Mechanical and Aerospace Engineering, The Ohio State University, Columbus, USA

## Abstract

Robotic lower limb prostheses can improve the quality of life for amputees. Development of such devices, currently dominated by long prototyping periods, could be sped up by predictive simulations. In contrast to some amputee simulations which track experimentally determined non-amputee walking kinematics, here, we explicitly model the human-prosthesis interaction to produce a prediction of the user’s walking kinematics. We obtain simulations of an amputee using an ankle-foot prosthesis by simultaneously optimizing human movements and prosthesis actuation, minimizing a weighted sum of human metabolic and prosthesis costs. The resulting Pareto optimal solutions predict that increasing prosthesis energy cost, decreasing prosthesis mass, and allowing asymmetric gaits all decrease human metabolic rate for a given speed and alter human kinematics. The metabolic rates increase monotonically with speed. Remarkably, by performing an analogous optimization for a non-amputee human, we predict that an amputee walking with an appropriately optimized robotic prosthesis can have a lower metabolic cost – even lower than assuming that the non-amputee’s ankle torques are cost-free.

People with trans-tibial (below knee) amputation commonly use passive prostheses providing some stiffness and damping (e.g., SACH-foot, Flex-foot^1^). However, passive prosthesis users experience reduced mobility^2^,^3^ and increased metabolic cost^4^,^5^ compared to non-amputees. This reduced performance is partially due to reduced foot control and an inability to produce net positive work with the prosthesis. Robotic ankle-foot prostheses could address these issues^6^,^7^. Although many robotic prostheses exist, designing and testing new prostheses takes considerable time: computational simulations could shorten this process. Many previous simulation studies^8^,^9^ used human-prosthesis models that tracked non-amputee walking kinematics, thus ignoring human adaptation to the prosthesis^10^. Here, rather than assume the amputee’s gait, we seek to predict it. Similarly, rather than assume a specific prosthesis actuation strategy, we design prosthesis torque profiles through energy optimization.

Numerous studies suggest that humans move in an approximately energy optimal manner^11^,^12^, even with amputation^13^ or using unpracticed motions^14^. Here, we use large-scale numerical optimization to compute the energy-optimal walking motions and prosthesis actuations of a human wearing a unilateral robotic prosthesis. We compute optimal trade-offs between human metabolic and prosthesis torque costs. We show how increasing prosthesis mass, using passive prostheses, or forcing left-right symmetry increase human costs for a given walking speed and how increasing speed increases cost. Finally, we predict that optimal prosthesis actuation can reduce the amputee metabolic cost much below normal human metabolic cost.

## Methods

### Human-prosthesis model

**Figure 1:**
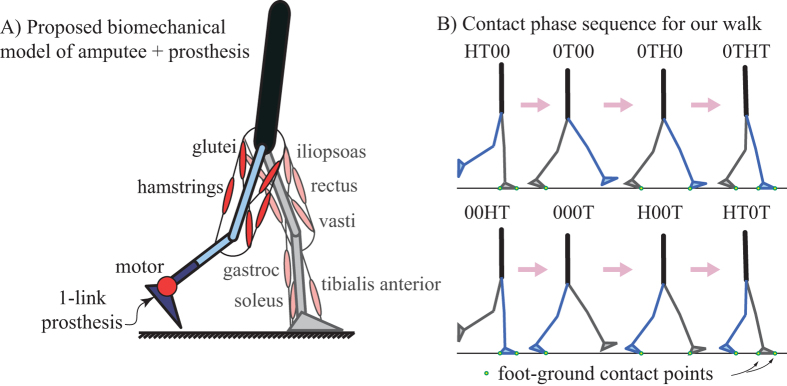
(**A**) A sagittal-plane rigid-body model, with six human body segments and one prosthesis segment, connected via revolute joints. The leg without amputation uses eight muscles (iliopsoas, gluteus, hamstring, rectus femorus, vastus lateralis, gastrocnemius, soleus, and tibialis anterior); the leg with amputation uses identical muscles but removing and replacing all muscles crossing the ankle with a single prosthesis torque motor at the ankle. (**B**) Contact phase sequence for a natural walking motion. The four letter code refers to the points contacting the ground; heel and toe of the biological and prosthetic foot with 0 signifying no contact.

We consider a sagittal plane model of a unilateral amputee using an ankle-foot prosthesis (Fig. 1A) with empirically-based properties^15^,^16^. The human component has six rigid-body segments: one head-arms-torso segment, one segment for each thigh, one segment for each shank, and one segment for the biological foot. There are thirteen uni- or bi-articular muscles with constant moment-arms and Hill-type force-velocity relationships: eight muscles on the biological (non-prosthesis) side and five on the prosthesis side. The prosthesis foot is a single rigid-body segment, actuated by an idealized torque motor with no internal dynamics. The prosthetic foot mass and inertia properties are identical to the human foot unless otherwise stated. See Supplementary Information for parameter values. We present all parameters and results in non-dimensional form, normalized by combinations of body mass *M*_body_, leg length 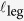, and gravitational acceleration *g*.

The feet can contact the ground at their heel and toe. We assume a normal walking motion with a prescribed sequence of eight ‘contact phases’ (Fig. 1B), each denoted by a four-letter code specifying which heels (H) and toes (T) are in contact or not (0): the first two letters refer to the biological foot’s contact points and the last two letters refer to the prosthesis’. e.g., HT0T has biological heel and toe and prosthesis toe in contact. Contact kinematic constraints are imposed using a differential-algebraic formalism, as opposed to modeling contact with springs and dampers^16^,^17^,^18^. By assuming that any point in contact with the ground has a zero velocity, this method of imposing contact constraints removes high frequency and fast time-scale dynamics that can arise due to springs and dampers. Given muscle forces and prosthesis torque as functions of time and initial conditions for body state, we can integrate the equations of motion for each contact phase to simulate the motion of the human-prosthesis system. We model transitions between contact phases as continuous when contact is broken and as perfectly-inelastic collisions, with velocity changing instantaneously when contact is made.

### Energy cost functions

We seek a periodic walking motion that minimizes 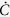, a weighted sum of the human metabolic cost *C*_met_ and the prosthesis cost *C*_pros_ over a stride as follows:


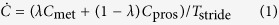


where *T*_stride_ is the stride time and 0 ≤ *λ* ≤ 1 is a fixed weighting factor: *λ* = 1 makes 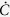 identical to human cost and *λ* = 0 gives the prosthesis cost. Optimizing with multiple *λ* values allows us to determine the trade-offs between human metabolic cost and prosthesis cost by changing the relative importance of the two costs in the optimization.

Our human metabolic cost function is adapted from Alexander and Minetti^11^,^19^:


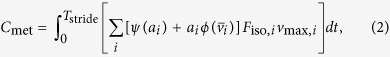


an integral over the whole stride period and a sum over all muscles, depending on activation *a*_*i*_, shortening velocity *v*_*i*_, maximum shortening velocity *v*_max,*i*_, and maximum isometric force *F*_iso,*i*_; *ψ*(*a*_*i*_) is an activation-related cost and 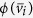 is a function^11^ describing the metabolic dependence on normalized muscle shortening velocity


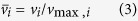


(see Supplementary Information, Figure S2). The prosthesis cost is modeled as


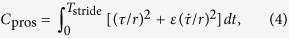


where *τ* is the motor torque, 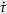 is the torque-rate, and *r* is a scaling constant (equal to typical muscle moment arm); the torque-squared term is a model of motor electrical losses^20^ and the torque-rate-squared term with a small pre-multiplier (*ε* = 0.01) is used to model torque production limitations by penalizing rapid activation and deactivation^11^,^21^.

### Other metabolic cost models

While all the results in the main manuscript are based on the above cost functions for the human and the prosthesis, we also considered two other simple cost functions that have previously been used in biomechanics to model “effort” or metabolic cost: (1) cost rate for each muscle proportional to muscle-force squared^16^,^22^, and (2) energy cost for each muscle proportional to a weighted sum of positive and negative work, scaled by the efficiencies of positive and negative work^11^,^23^. We did not use other more complex models based on muscle heat generation^18^,^24^, as they are often non-smooth; we will consider such functions in future work.

### Optimization structure and constraints

We seek an optimal walking gait with the periodic contact phase sequence in Fig. 1B, parameterized using a “multiple-shooting method”. We constrained speed at *v* = 1.3 m/s or non-dimensional speed 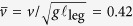, except for one set of calculations that varied speed systematically. The eight contact phases, *N*_phases_ = 8, each with unknown time duration, are divided into *N*_seg_ = 6 time-segments, each with *N*_unknowns_ = 32 unknowns (initial conditions for all body segment positions and velocities and unknown muscle forces and prosthesis motor torque, assumed piecewise linear), producing 1544 unknowns through:


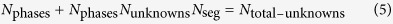


to be determined by optimization. We constrain positions, velocities, forces and torques to be continuous across different time segments. For collisional transitions, the results of the collision equations (applied to the end of the contact phase) are equal to the initial conditions for the next contact phase. Further nonlinear constraints include ground clearance for swing legs, ground contact for stance legs, muscle force-velocity dependence, and range of motion bounds. We solve the constrained optimization problem using sparse nonlinear programming (SNOPT^11^), with equality constraint satisfaction of 10^−7^. Since it is possible for the optimization to converge to non-global local minima, we only consider solutions which can be discovered from multiple initial seeds.

## Results

### Optimizing just the human cost (mostly)

**Figure 2:**
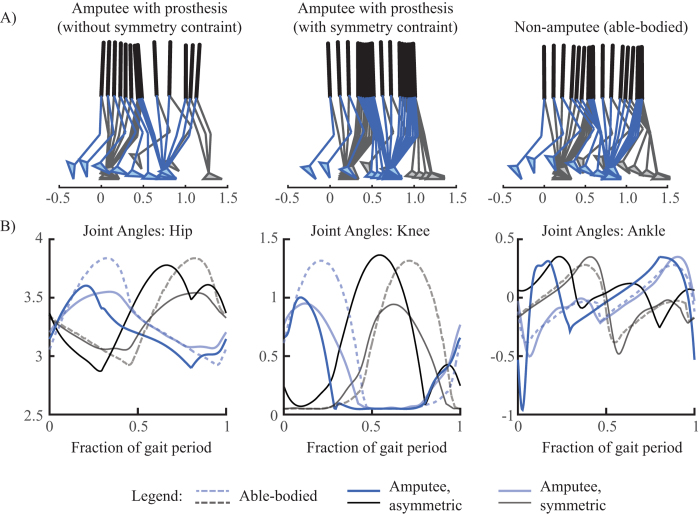
(**A**) Optimal gait kinematics for amputee walking (with and without symmetry constraint) with *λ* = 0.95 and non-amputee walking. For the amputee, the blue leg represents the prosthesis and the black leg represents the biological one. (**B**) Optimal joint angles for all three conditions over one gait cycle with periods of 2.876 (asymmetric), 2.412 (symmetric), and 2.857 (non-amputee). See Supplementary Video V1 for a video animation of these optimized walking motions.

We minimized the composite cost 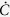 strongly weighted towards human cost (*λ* = 0.95), to obtain optima when prosthesis cost is mostly neglected. Figure 2A,B and the walking animations (Supplementary Video V1) show the corresponding optimal motion is asymmetric.

Setting *λ* = 1 caused convergence issues in the numerical optimization; presumably because this limit *λ* = 1 implies a truly “zero cost prosthesis,” the optimization tended to explore large and erratic prosthesis motor torques and torque rates, resulting in the numerical difficulties.

### Comparison with non-amputee gait

**Figure 3:**
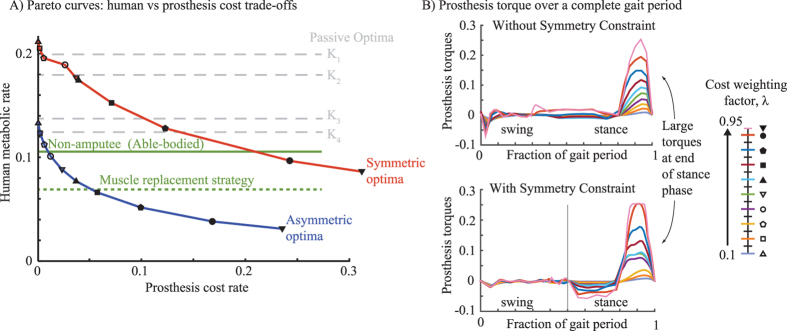
(**A**) Optimal trade-offs (Pareto curves) between human and prosthesis costs: without symmetry constraint (blue line) and with symmetry (red line). Different markers (legend) denote results from different *λ*’s (0.1–0.95). Optima from passive prostheses are shown for four non-dimensional stiffnesses (gray dashed line, K_1_ = 0.1, K_2_ = 0.5, K_3_ = 1.0, K_4_ = 1.5), as are non-amputee optimum (green line) and the non-amputee optimum (green dotted line) with muscle replacement strategy (cost-free ankle muscle costs). See Figure S3 (Supplementary Information) for analogous Pareto trade-off curves for two other metabolic cost models. (**B**) Prosthesis torque over one stride with gait symmetry unconstrained and constrained. Each line represents an optimization with different *λ* (0.1–0.95). The labels ‘swing’ and ‘stance’ are left without a clear demarcation of when these phases occur, because they are different durations for the different optimal gaits when asymmetry is allowed; with symmetry, each of these phases is 50% of the gait period.

We minimized the same human metabolic cost for a non-amputee (able-bodied) human with symmetric legs and musculature, giving a symmetric optimal gait (Fig. 2A,B). Metabolic cost for the amputee with a prosthesis is lower than that for the non-amputee (Fig. 3A). The muscles crossing a single ankle contribute 41% of the optimized non-amputee metabolic cost. Thus, if ideal motor torques replaced all muscles crossing one ankle while maintaining identical kinematics, the human cost could be reduced by 41% by this “muscle replacement strategy”. Remarkably, for *λ* = 0.95, the human cost with a unilateral prosthesis is 73% lower than the non-amputee cost. This greater cost reduction arises from allowing amputee kinematics to be different from non-amputee kinematics, thus allowing the prosthesis ankle motor to perform much more work than the replaced ankle muscles. This is remarkable because while the replaced ankle muscles provide torques at both ankle and knee (some muscles are biarticular), our prosthesis produces torques only at the ankle. Thus, extrapolating this trend, we speculate that if we made the prosthesis provide torques at the knee (simulating a supplementary knee exoskeleton) as well as at the ankle, perhaps the optimal cost reduction might be even greater.

### Optimal trade-offs between human and prosthesis cost

By optimizing with different *λ*’s between 0.1 and 0.95, we obtain the optimal cost trade-off between human and prosthesis costs (Fig. 3A). This trade-off curve, often called a “Pareto curve”^25^, shows that increasing prosthesis cost decreases human cost. Here, even though obtained by minimizing a weighted sum of human and prosthesis cost, the Pareto curve (if ‘convex’) also has the following interpretation: this curve gives the lowest human cost for a given prosthesis cost and vice versa. Any other walking strategy will have either a higher human cost or a higher prosthesis cost compared to every point on this Pareto curve. Figure 3B shows that the the optimal prosthesis actuation has most of prosthesis action at the end of stance phase, providing large push-off power; the human cost is reduced through an increase in prosthesis push-off torque and impulse. While the trade-off in Fig. 3A is specific to the assumed human and prosthesis costs, we find that almost identical trade-off curves arise when substantially different cost functions are used (Figure S3, Supplementary Information).

### Symmetry is expensive

Every Pareto-optimal gait we found was asymmetric irrespective of *λ*. However, walking symmetry is said to have physiological and psychological benefits to prosthesis users^26^, so we repeated the optimizations while requiring approximate left-right symmetric kinematics. Symmetry was enforced as a constraint requiring the left leg joint angles and angular rates during one step be nearly equal to (within 0.05 rad or 0.05 rads^−1^ of) the right leg joint angles and angular rates one step later. The resulting symmetric optimal gaits had much higher human and prosthesis costs (Fig. 3A) for each *λ*, a longer push-off phase, and different timing for dorsiflexion torques during swing phase (Fig. 3B). When we compared the cost of the optimal symmetric gait at *λ* = 0.95 to the non-amputee condition, we found only a 27% reduction in the metabolic cost. This reduction in cost is smaller than the 41% metabolic cost reduction achieved by the replacement strategy. The optimal symmetric gait with the prosthesis is worse than the simple replacement strategy, even though the replacement strategy would also result in a symmetric gait; while we do not know the reason for these relative costs, presumably the optimal symmetric gait with the prosthesis has a higher cost because it is unable to replace the knee torques with the prosthesis (no Gastrocnemius), requiring other muscles to compensate.

### Lighter feet are less expensive

**Figure 4:**
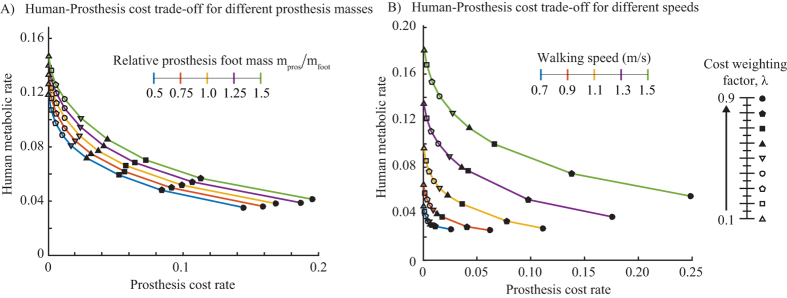
(**A**) Pareto curves between human and prosthesis cost for five different prosthetic foot masses. Lower prosthetic foot mass is better. (**B**) Pareto curves with five different walking speeds. Lower speeds have lower costs.

We performed optimizations with prosthetic foot masses from 50%–150% that of the intact foot, scaling the prosthesis moments of inertia similarly. Figure 4A shows that by fixing either the human or the prosthesis cost, we can reduce the other by reducing prosthesis mass. Further, we see that cost reductions from reducing foot mass are much smaller than those obtained by increasing the prosthesis cost. Both human and prosthesis costs seem well-approximated by a linear dependence on the prosthesis mass for each *λ* (Figure S4, Supplementary Information), but the coefficients of the linear fit depends on *λ*.

### Greater human cost reduction at higher speeds

We performed optimizations with five different walking speeds between 0.7 and 1.5 m/s, computing the Pareto curves with *λ* = 0.1–0.9. As is true for non-amputee walking, the human metabolic rate increases with increasing speed (Fig. 4B). In particular, the whole Pareto curve for a lower speed is below and to the left of that for a higher speed, implying that for a given Prosthesis cost rate, a lower speed implies a lower human metabolic rate and vice versa. Further, we see that for lower speeds, we predict lower percentage and absolute reduction to the human metabolic rate while using an optimal active prosthesis with most human benefit (here *λ* = 0.9).

### Passive prosthesis

We performed optimizations constraining the prosthesis to be a linear torsional spring and damper, simulating a passive prosthesis. We considered four stiffnesses: 0.10, 0.50, 1.00 and 1.50 non-dimensional stiffness normalized by bodyweight, chosen to roughly capture the torque-angle relationship of optima derived earlier. Damping was chosen to make the foot over-damped during swing; we used 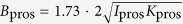, where *B*_pros_ is the damping, *I*_pros_ is the prosthesis moment of inertia, *K*_pros_ is the torsional stiffness, and 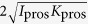 is the critical damping value for a second order linear system. While the metabolic cost decreases as the stiffness increased, all of these passive devices produced asymmetric and metabolically expensive human gaits (Fig. 3A). Human cost with the passive device is comparable to active prosthesis with a symmetry constraint, except for high *λ*’s. Thus, the symmetry constraint is so detrimental as to make active robotic prostheses have little to no energetic benefit over passive devices.

## Discussion

We have obtained the optimal tradeoffs between human and prosthesis costs for a robotic unilateral prosthesis, and have suggested that we can reduce amputee metabolic cost by increasing prosthesis effort, allowing asymmetry and decreasing prosthesis mass. These relationships can inform prosthesis design, e.g., by selecting a desired metabolic cost, we could predict the prosthesis cost at various prosthesis masses; the prosthesis torques and costs along with information on number of steps walked daily will allow us to pick motor and battery specifications for the prosthesis. We have created a design tool that translates a specification of the level of assistance to be provided by the prosthesis (namely, *λ*) into appropriate prosthesis torque profiles.

We have not compared predicted human kinematics and performance with experiment. Such comparison requires implementing our optimal actuation in a prosthesis; a simpler comparison might involve using a passive prosthesis in experiment and comparing with the corresponding model-derived optimal human kinematics. Highly accurate prediction of even non-amputee walking kinematics in a wide variety of novel situations remains an open problem; recent attempts have fallen short of quantitatively predicting the correct kinematics, kinetics, and/or metabolic costs^16^,^17^,^18^, even though having qualitatively similar kinematics, analogous to our non-amputee optimization here. Improved predictions of human walking with a prosthesis may require more complex human body models, more accurate metabolic cost models, and inclusion of other objectives such as lowered interaction forces between human and prosthesis.

Our optimization predicts asymmetric gaits for both robotic and passive prostheses but the mechanism that causes the asymmetry is likely different in these two settings. When using a passive prosthesis, the gait becomes asymmetric since the prosthesis cannot add positive work to the system and the human is compensating with the intact limb. However, for robotic prostheses, our model suggests asymmetric gaits are energy optimal with symmetric gaits costing vastly more. In these asymmetric optimal gaits, the leg with the prosthesis spends more time on the ground (higher duty factor, Figure S5, Supplementary Information) compared to the biological leg, allowing the robotic prosthesis to provide greater assistance; we predict that optimal prosthesis actuation could reduce the human cost, possibly much below non-amputee levels (by over 70%). This energy reduction prediction is considerably higher than observed in experimental studies, which have at best reduced the users’ metabolic costs to about equal to non-amputee metabolic cost^27^ (about 14% reduction compared to the subjects’ passive prostheses).

This discrepancy in energy costs is likely because our predictions are based on simultaneous optimization of human and prosthesis control and therefore a best case scenario, whereas the controllers in current robotic prostheses are not generally optimized to the person and the person may also not have had enough time to adapt to the prosthesis. Our optimal prosthesis actuation as a function of time has a large torque impulse near the end of the stance phase; it may be that current robotic prostheses, with their simpler ankle-state-based feedback controllers, are unable to allow the prosthesis to produce a large torque impulse right at the end. Most current prosthesis also have a lower peak torque than allowed in our simulations, as our peak torque was based on a recent physical prosthesis emulator^28^, reported as having the higher torque output among current prostheses. Further, whereas our prosthesis actuation is specific to walking at a particular speed on level ground, the feedback controllers in current robotic prostheses may have been the result of compromises made for use at different walking speeds, slopes, etc. Producing large energy reductions in experiment may require a prosthesis capable of optimizing its torque output to the person and the environment (perhaps over a training period). Other issues that compound the discrepancy in costs between current prostheses and our predictions include secondary goals sought by the user (e.g. gait symmetry, pain reduction, lateral-stability, etc.), or other un-modeled effects.

Alternatively, it may be that the large energy reduction we predict is due to our specific metabolic cost model. Experimental studies in non-amputees suggest that each ankle contributes about 13% of the metabolic cost of walking^29^ while our metabolic cost model predicts a total of 41%. Repeating the non-amputee calculation with a work-based metabolic cost, we found that only about 18% of the total work cost is due to one ankle – this is the reduction predicted by a muscle replacement strategy for this work-based cost (Figure S3 and Table S5, Supplementary Information). For this work-based cost, the robotic prosthesis (with *λ* = 0.95) produced only a 23% reduction in cost, still greater than the 18% from the muscle replacement strategy, but much lower than 73%. On the other hand, for a scaled muscle-force-squared cost, the ankle contribution was about 43% for a non-amputee walk and the reduction from the robotic prosthesis was about 74% (Figure S3 and Table S5, Supplementary Information). Thus, it appears that some of these specific numerical predictions may rely on the metabolic cost model and may be improved with a much more accurate metabolic cost model, which remains an open problem^11^,^18^.

Having performed over a hundred different optimization calculations under different parameter conditions (in contrast to other optimization-based studies^16^,^17^,^18^), we have demonstrated feasibility of using such large-scale optimizations in a formal design procedure with user-specific model parameters. Future work could consider the effects of different prosthesis structures (including non-ideal actuators and passive elements) and constraining the prosthesis torque to be a function of system state rather than be allowed to vary arbitrarily in time: for instance, by using existing controller structures found in other prostheses or by parameterizing a torque versus ankle angle relation for a gait and optimizing the controller parameters. Such additions will allow us to compare the results of our simulation to those found through experiment, and improve the model to make accurate quantitative predictions.

## Additional Information

**How to cite this article**: Handford, M. L. and Srinivasan, M. Robotic lower limb prosthesis design through simultaneous computer optimizations of human and prosthesis costs. *Sci. Rep.* **6**, 19983; doi: 10.1038/srep19983 (2016).

## Supplementary Material

Supplementary Video S1

Supplementary Information
