# Supplementary material for: Robotic lower limb prosthesis design through simultaneous computer optimizations of human and prosthesis costs
Source: Sci Rep. 2016 Feb 9;6:19983. doi: 10.1038/srep19983 (PMC4746571; doi:10.1038/srep19983)
Supplement: Supplementary Information [file srep19983-s2.pdf]

Supplementary Information Appendix for:

Robotic lower limb prosthesis design through simultaneous computer optimizations of human and prosthesis costs

Matthew L. Handford and Manoj Srinivasan

Mechanical and Aerospace Engineering, The Ohio State University, Columbus, USA

handford.4@osu.edu, *srinivasan.88@osu.edu*

Note. The tables and figures in the supplementary information are numbered S1, S2, etc., whereas those in the main manuscript are numbered 1, 2, etc. This Supplementary Information Appendix provides all model parameters and other technical information relevant to the optimization. In addition, there is a supplementary video V1 available online, the caption/legend for which is repeated below.

Supplementary Video V1. Animations of optimized human walking with a robotic prosthesis (without and with a symmetry constraint), human walking with a passive prosthesis, as well as an optimized non-amputee walk. For the amputee motions, we use blue for the leg with the prosthesis and gray for the leg without the prosthesis.

S1 Parameters

The schematic (Figure S1) shows the axis conventions for the positions of the center of masses (CoM) and other key body locations.

The biped model parameters provided in Tables S1-S3 follow those in Gerritsen et al and van den Bogert tutorial [1, 2]. (The parameters are drawn directly from the van den Bogert tutorial [2], stated to be the same parameters as in Gerritsen et al [1]; Gerritsen et al [1] do not actually specify the parameter values.) The

Alexander-Minetti-like metabolic cost dependency on muscle activation and muscle shortening velocity are shown in Figure S2 and its caption.

S2 Other objective functions

We chose to check the results found using the metabolic cost function described in the Methods section of the main document and Figure S2 with two other common objective functions. The first of these objective functions is a scaled force-squared and torque-squared cost function defined by:

$$\dot{C} = \frac{1}{T_{\text{stride}}} \int_0^{T_{\text{stride}}} \left[\lambda \sum_i \frac{F_i^2}{F_{\text{iso}}^2} \cdot F_{\text{iso}} v_{\max} + (1-\lambda)(\tau/r)^2 \right] dt \tag{1}$$

where the sum is over all muscles, F_i is the force produced by the ith muscle, τ is the prosthesis motor torque, r is a scaling constant (equal to typical muscle moment arm), T_{stride} is the total time of one stride, λ is the weighting factor, and the product $F_{\text{iso}} v_{\max}$ has units of power and provides appropriate scaling of the cost for various muscles as in [3]. The second objective function is a work based cost defined by:

$$\dot{C} = (\lambda \sum_i (4W_{\text{m},i}^{+} + 0.83W_{\text{m},i}^{-}) + (1-\lambda)|W_{\text{pros}}|)/T_{\text{stride}} \tag{2}$$

where the sum is over all muscles, $W_{\text{m},i}^{+}$ is the positive muscle work, $W_{\text{m},i}^{-}$ is the negative muscle work, and W_{pros} is the prosthesis work over the stride. Using either of these objective functions produced the same qualitative results in terms of energetic trade off and kinematic changes as those found with the metabolic cost function. We compared the costs of the prosthesis with those from the non-amputee tests with each objective function. The results of this analysis can be observed in table S5. The Pareto curves from each of the three objective functions used for this research are displayed in Figure S3.

S3 Cost dependence on prosthesis mass

After completing tests with prosthesis mass equal to 0.5 to 1.5 times the mass of the intact foot, we found a positive correlation between mass and both human and prosthesis costs. Moreover these cost rates depend

linearly on mass for a given λ. Figure S4 displays the linear dependence for all λs tested.

References

[1] K. G. Gerritsen, A. J. van den Bogert, M. Hulliger, and R. F. Zernicke. Intrinsic muscle properties facilitate locomotor control - a computer simulation study. *Motor Control*, 2:206–220, 1998.

[2] A. J. van den Bogert. Gait2de – a musculoskeletal dynamics model for posture and gait. In *Conference on Dynamic Walking, Jena*, 2011.

[3] A.E. Minetti and R. McN. Alexander. A theory of metabolic costs for bipedal gaits. *J. Theor. Biol*, 186:467–476, 1997.

[4] M. Srinivasan. Fifteen observations on the structure of energy minimizing gaits in many simple biped models. *Journal of the Royal Society Interface*, 8:74–98, 2011.

[5] Roger C Woledge, Nancy A Curtin, and Earl Homsher. Energetic aspects of muscle contraction. *Monographs of the physiological society*, 41, 1985.

[6] M. Srinivasan. Optimal speeds for walking and running, and walking on a moving walkway. *Chaos: An Interdisciplinary Journal of Nonlinear Science*, 19:026112, 2009.

[7] M. Srinivasan and A. Ruina. Computer optimization of a minimal biped model discovers walking and running. *Nature*, 439:72–75, 2006.

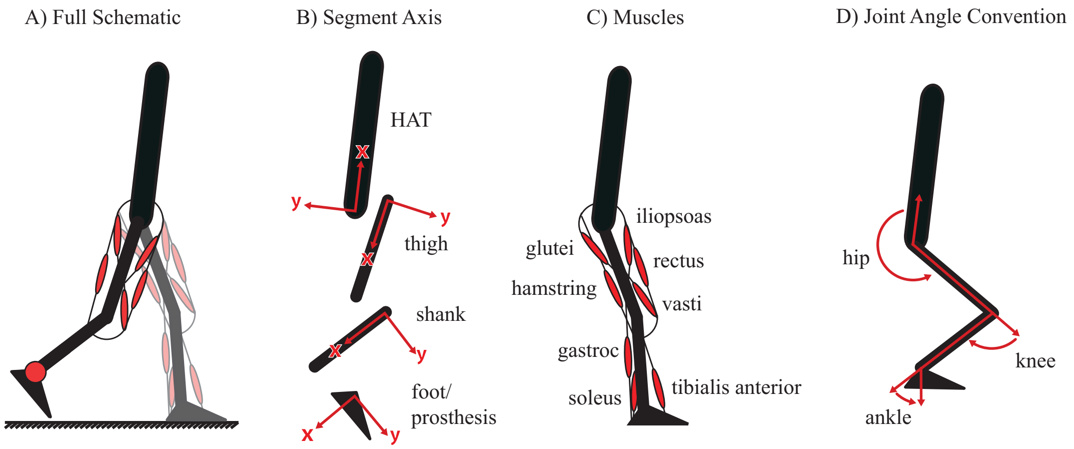

Figure S1: Biped model. A) A basic representation of the sagittal-plane human-prosthesis model. B) Origin and axis information for each of the segments. C) Graphical representation of the muscle groups. D) Joint angle convention based on rotation of relative axes with the positive direction corresponding to flexion/dorsiflexion of the joints.

Segment	Mass, kg	Moment of Inertia, kg m^2	x CoM, m	y CoM, m
HAT	50.85 (0.6879)	3.177 (47.25e-4)	0.3155 (0.3308)	0 (0)
Thigh	7.5 (0.1015)	0.1522 (22.63e-4)	0.1910 (0.2003)	0 (0)
Shank	3.49 (0.0472)	0.0624 (9.280e-4)	0.1917 (0.2010)	0 (0)
Foot	1.087 (0.0147)	0.0184 (2.736e-4)	0.0351 (0.0368)	0.0768 (0.0805)
Prosthesis	1.087 (0.0147)	0.0184 (2.736e-4)	0.0351 (0.0368)	0.0768 (0.0805)

Table S1: Biped inertia parameters. The center of mass distances are measure from the origin of the segment connected proximal joint (Figure S1b). The x distance is along the segment while y is perpendicular to the segment. Values in parentheses show the corresponding dimensionless quantity. The moment of inertia are about the z axis (perpendicular to sagittal plane), through the center of masses of the respective segments. The mass and moment of inertia for the prosthesis correspond to the standard condition where $m_{\text{pros}} = m_{\text{foot}}$

Segment	x Distance, m	y Distance, m
HAT	0.6 (0.6290)	0 (0)
Thigh	0.4410 (0.4624)	0 (0)
Shank	0.4428 (0.4642)	0 (0)
Heel (bio/pros)	0.07 (0.0734)	-0.06 (-0.0629)
Toe (bio/pros)	0.07 (0.0734)	0.15 (0.1573)

Table S2: Biped segment length and foot shape parameters. For the HAT, thigh and shank segments, the segment lengths are measured along the x axis from the origin of the segment, which is at the proximal joint (Figure S1b), to the distal joint; for these three segments, the y distance between the joints is zero, as seen in the first three rows of the table. For the foot, we show the vector from the ankle to the heel and the toe. Values in parentheses show the corresponding dimensionless quantity.

Muscle	F_{iso}, N	v_{max}, m/s	k_{tendon}, N/mm	d_{hip}, mm	d_{knee}, mm	d_{ankle}, mm
iliopsoas	1500 (2.068)	1.069 (0.3334)	264.1 (347.4)	50 (52.42)	0 (0)	0 (0)
glutei	3000 (4.137)	2.097 (0.6538)	477.7 (628.4)	-62 (-65.00)	0 (0)	0 (0)
hamstrings	3000 (4.137)	1.090 (0.3400)	224.6 (295.4)	-72 (-75.48)	-34 (-35.64)	0 (0)
rectus	1200 (1.655)	0.8492 (0.2648)	75.37 (99.15)	34 (35.64)	50 (52.42)	0 (0)
vasti	7000 (9.653)	0.9750 (0.3040)	784.8 (1032)	0 (0)	42 (44.03)	0 (0)
gastroc	3000 (4.137)	0.5766 (0.1798)	178.6 (234.9)	0 (0)	-20 (-20.97)	-53 (-55.57)
soleus	4000 (5.516)	0.5766 (0.1798)	408.2 (536.9)	0 (0)	0 (0)	-53 (-55.57)
tibialis anterior	2500 (3.448)	0.8597 (0.2681)	197.2 (259.3)	0 (0)	0 (0)	37 (38.79)

Table S3: Biped muscle parameters. A table displaying the max isometric force F_{iso}, max contractile velocity v_{max}, tendon stiffness k_{tendon}, and moment arm d at each joint for all 8 muscle groups. The moment arms marked as zero indicate that the muscle does not cross that joint. These muscle groups are shown graphically in Figure S1c. We ignore the muscles' force-length dependence conventional in Hill-type muscle models. The prosthesis torque is bounded by 175 Nm (0.253 non-dimensional). All parameters from [1, 2]. We use a linear force velocity relation, defined by the two parameters F_{iso} and v_{max}.

Joint	Angle lower bound	Angle upper bound
Hip	1.571	6.2831
Knee	0.05	1.885
Ankle	-0.9599	0.3491
Prosthesis	-0.9599	0.3491

Table S4: Biped joint ranges of motion. The joint angles are displayed graphically in Figure S1d. This table presents the bounds on these angles (in radians). Some bounds are assumed to be larger than anatomical, but such bounds are never active at the optimal solution, as is clear from the optimal kinematics depicted in the main manuscript (Figure 2).

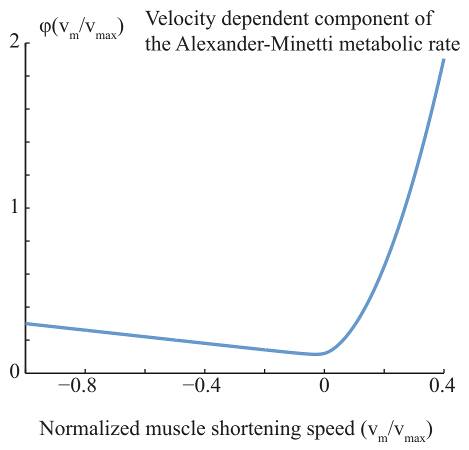

Figure S2: Metabolic cost details. The metabolic cost expression adapted from [3, 4], has two terms: an activation cost ψ and a muscle-shortening related term ϕ. The muscle-shortening related term ϕ, depicted in this figure, is based on empirical heat and ATPase activity [3] and approximated by $\phi = 0.1 + 0.9(\bar{v})^+ + 0.2(\bar{v})^- + 9(\bar{v})^+(\bar{v})^+$, where $\bar{v}$ is the muscle contractile velocity over the maximum contractile velocity, $(\bar{v})^+$ is the positive part of the $\bar{v}$, and $(\bar{v})^-$ is the negative part of $\bar{v}$. This function is used to model the differing metabolic cost between a contracting muscle and an extending muscle. See [4] for more details. The activation cost $\psi(a_i) = 0.05\left(a_i + a_i^2\right)$ is a function that captures the empirical result that about 40% of isometric muscle exertion cost is the activation cost [5]. Because we optimize for a fixed walking speed, we do not include a resting cost to the total metabolic cost. Adding a fixed resting metabolic rate will simply add a constant term to all the metabolic costs and does not change any of the optimal strategies [6].

Objective function	Non-amputee	Replacement strategy (% reduction)	Amputee (% reduction)
Alexander-Minetti	0.1054	0.0622 (41%)	0.0312 (73%)
Force-squared	0.0547	0.0319 (42%)	0.0143 (74%)
Work-based	0.0437	0.0357 (18%)	0.0335 (23%)

Table S5: Other objective functions. The non-dimensional human cost using Alexander-Minetti, Force-squared, and work-based costs.

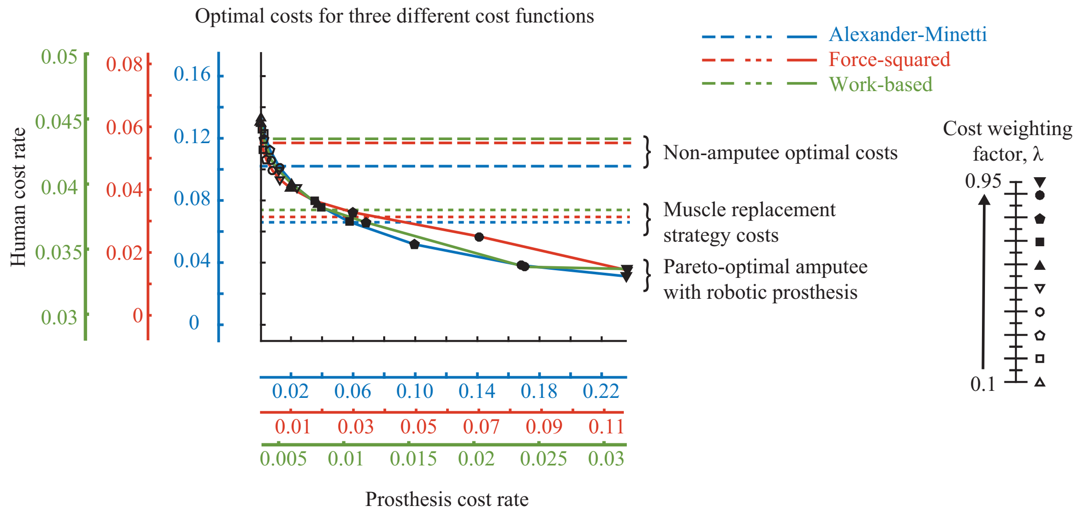

Figure S3: Pareto curves for two other cost models. Pareto curves for three different cost functions are shown (solid curves): (1) using Alexander-Minetti cost for human and smoothed torque-squared cost for prosthesis, as in the main manuscript (Figure 3A), (2) using a scaled muscle-force-squared cost for the human and a scaled torque-squared cost for the prosthesis, (3) using a muscle work-based cost for the human and a motor work cost for the prosthesis. Thus, these Pareto curves show that all these different costs give qualitatively similar trade-offs between human and prosthesis energy costs for the amputee. The work based costs produced impulsive muscle forces and prosthesis torques, as also observed in earlier work [4, 7]. For the three cost functions, we also show the optimal costs for an able-bodied (non-amputee) walker (long-dashed line) and that for the "muscle replacement strategy" (short-dashed line), demonstrating that the optimal robotic prosthesis actuation reduces amputee energy cost below both the able-bodied walker and an able-bodied walker with cost-free muscles crossing an ankle.

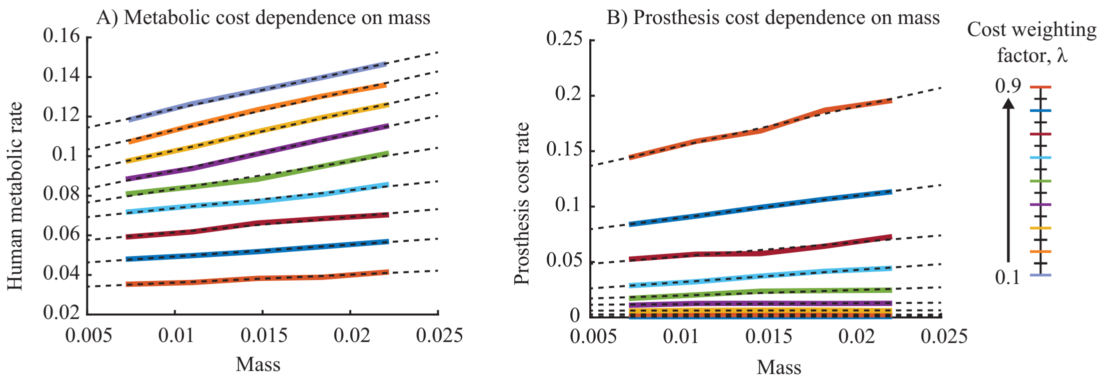

Figure S4: Linear dependence of cost on prosthetic foot mass. Linear-dependence on mass of (A) human metabolic cost rate and (B) prosthesis cost rate.

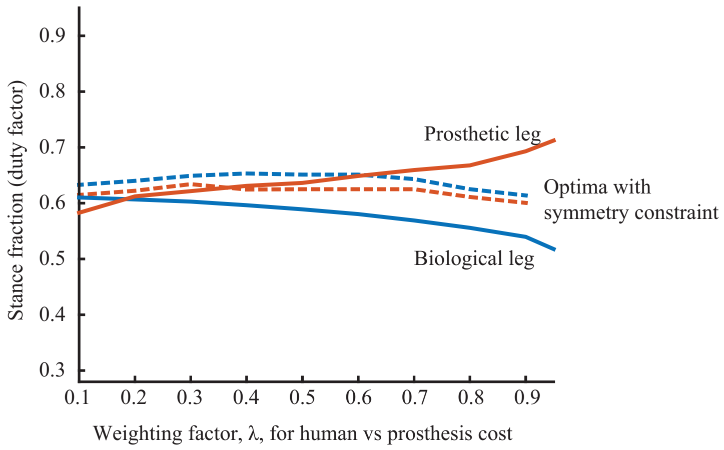

Figure S5: Duty factor. Duty factor (a leg's stance duration as a fraction of total stride period) is shown for the two legs for the Pareto-optimal amputee walking, as a function of the weighting factor λ. When asymmetric gaits are allowed (solid lines), we find that the prosthetic leg spends more time in stance than the biological foot as λ is increased, presumably to allow the prosthesis to provide more assistance. When we have symmetry constraints (dashed lines), we find that the two legs have slightly different stance periods because the symmetry constraint does not impose perfect symmetry.
